# Supplementary material for: Doxorubicin Loading into Milk and Mesenchymal Stem Cells’ Extracellular Vesicles as Drug Delivery Vehicles
Source: Pharmaceutics. 2023 Feb 21;15(3):718. doi: 10.3390/pharmaceutics15030718 (PMC10051717; doi:10.3390/pharmaceutics15030718)
Supplement: Supplementary file 1 [file pharmaceutics-15-00718-s001.zip › pharmaceutics-2057933-supplementary.pdf]

Supplementary information

Supplementary Figure S1

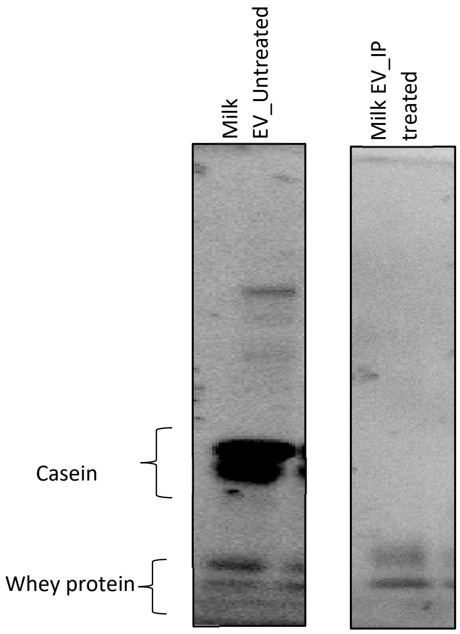

Supp Figure S1: Total protein separation by polyacrylamide gel electrophoresis indicates efficient removal of casein proteins in IP treated milk samples compared to untreated milk samples.

Supplementary Figure S2

20 µg Dox

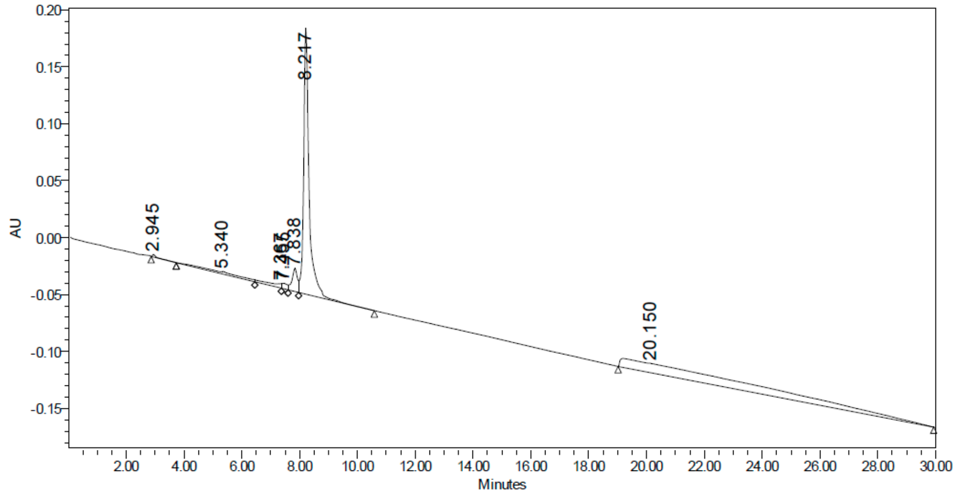

10 µg Dox

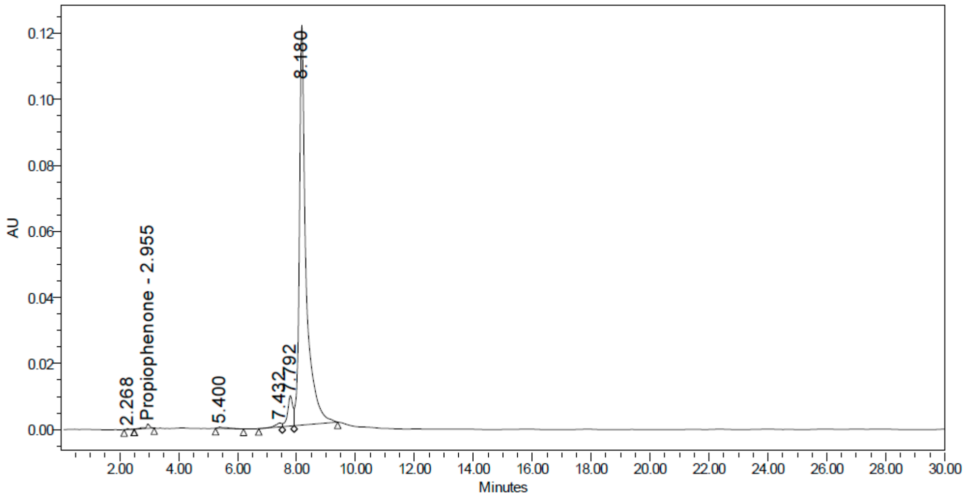

5 µg Dox

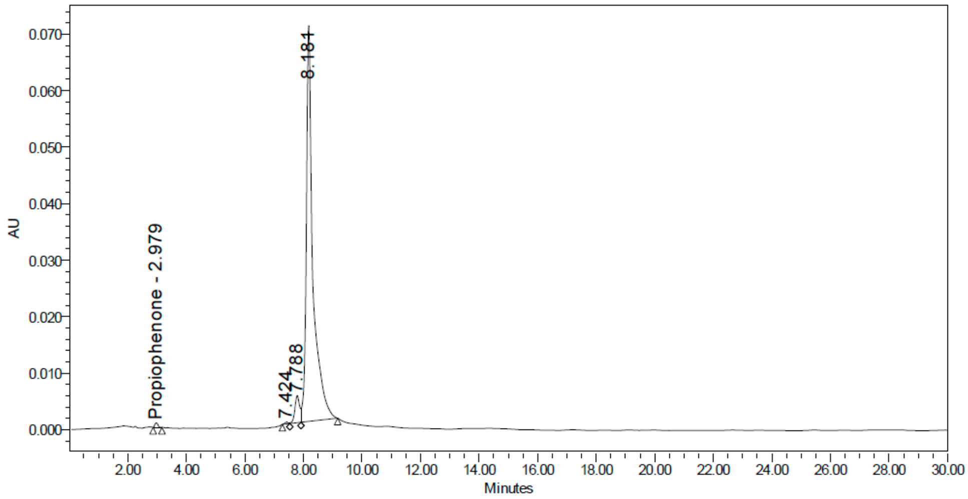

2.5 µg Dox

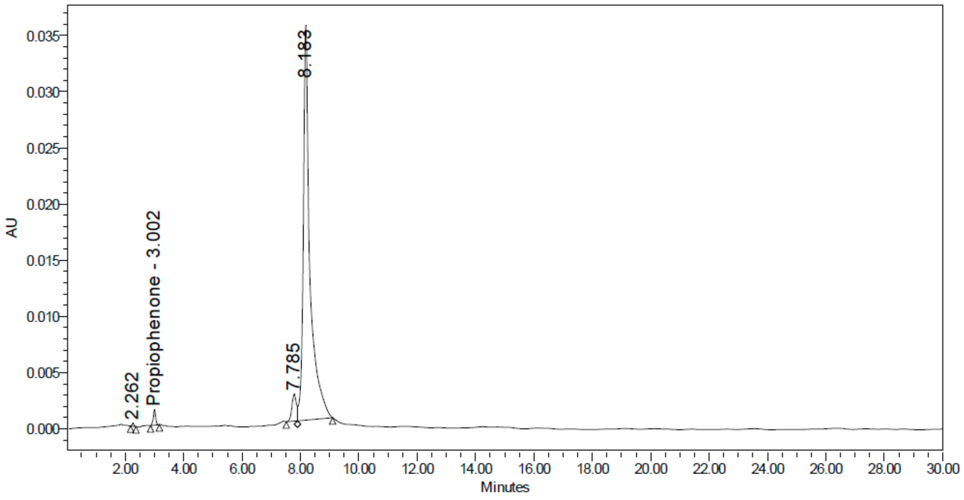

Dox Standard curve

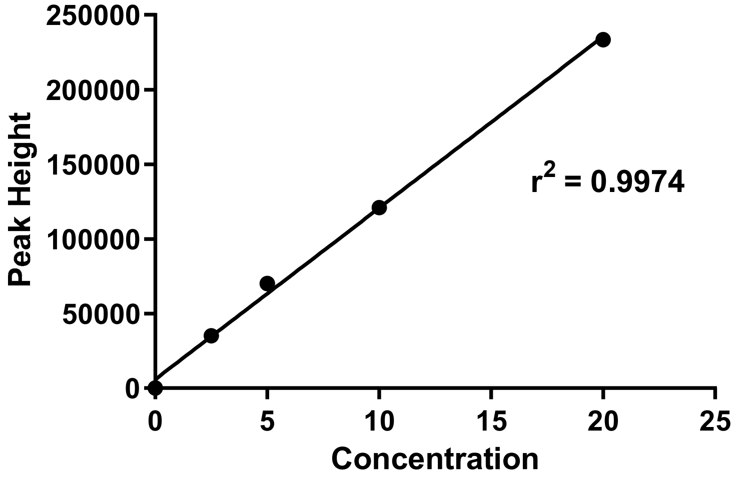

Supp Figure S2a: Serially diluted Dox samples were analysed by HPLC to obtain the standard curve and unknown concentration of Dox from loaded EV samples were calculated using the straight-line equation. Interpolation of unknown Dox was performed on GraphPad Prism 9 software.

(**b**) Representative Dox peaks obtained by HPLC

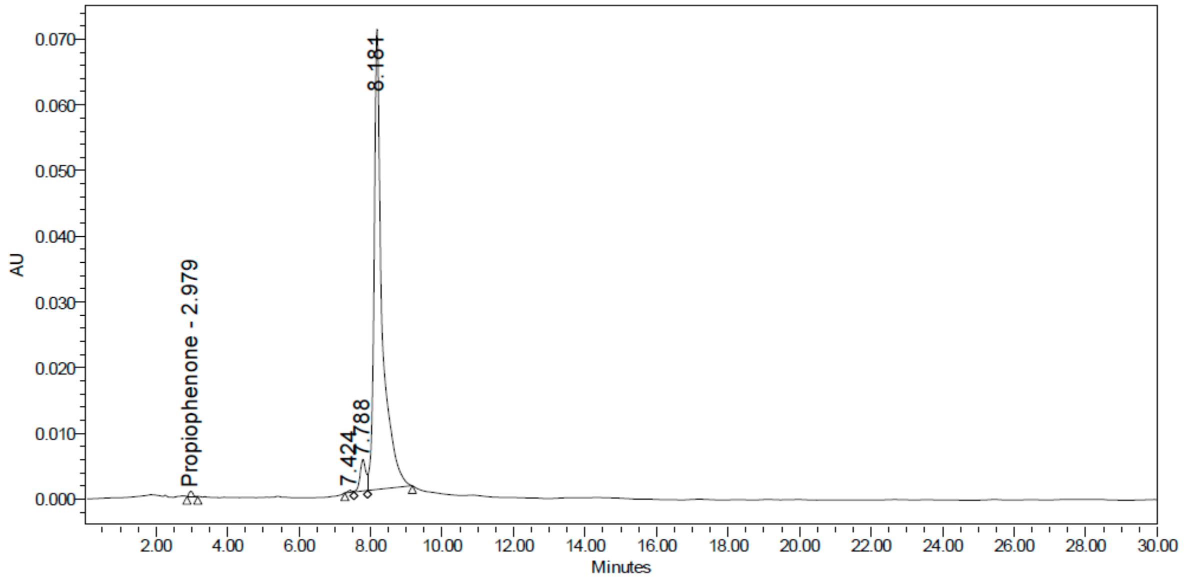

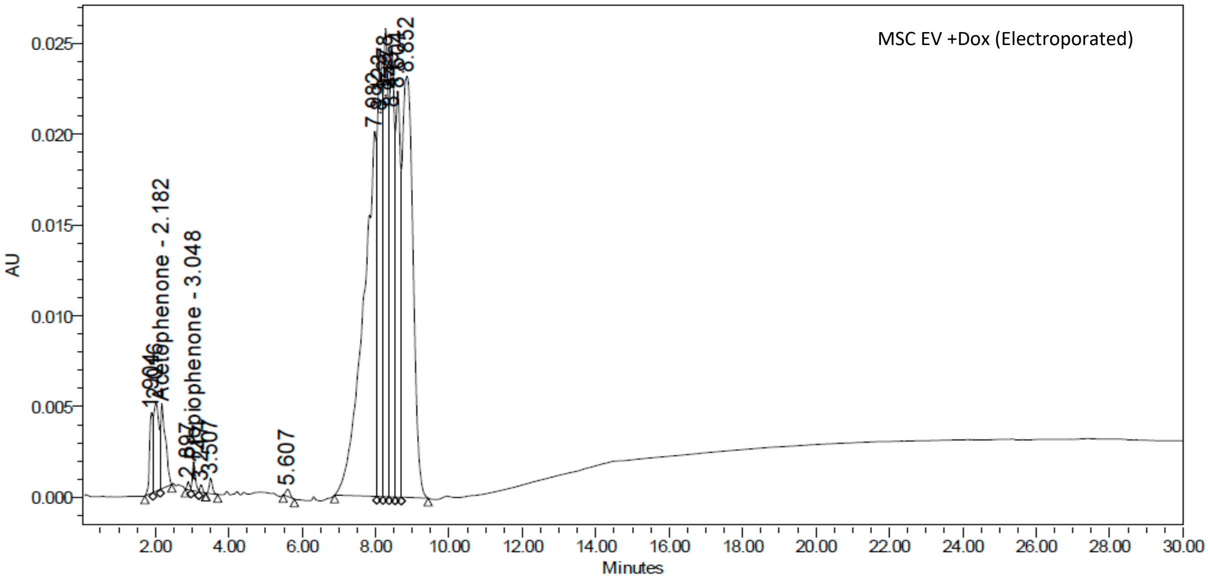

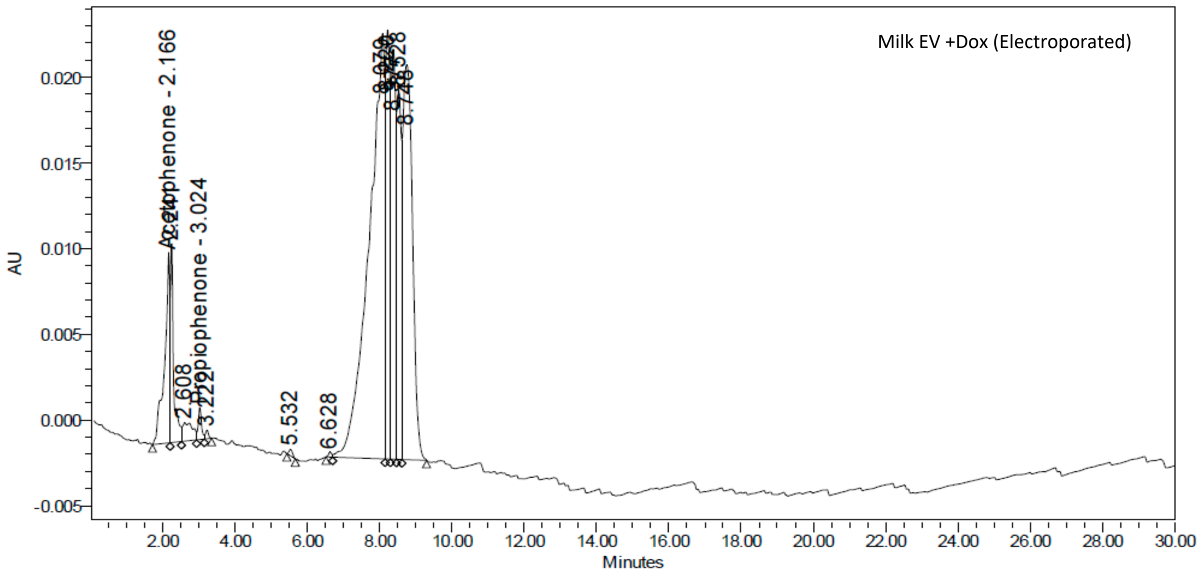

Supp Figure S2b: Representative doxorubicin peaks were obtained by HPLC analysis of Dox alone and Dox-loaded MSC EVs and milk EVs (lysed).
